# Towards a comprehensive model for understanding adaptations’ impact: the model for adaptation design and impact (MADI)

**DOI:** 10.1186/s13012-020-01021-y

**Published:** 2020-07-20

**Authors:** M. Alexis Kirk, Julia E. Moore, Shannon Wiltsey Stirman, Sarah A. Birken

**Affiliations:** 1 The Impact Center, Frank Porter Graham Child Development Institute, The University of North Carolina at Chapel Hill, 105 Smith Level Road, Chapel Hill, NC 27599 USA; 2 The Center for Implementation, 20 Northampton Dr., Toronto, ON M9B 4S6 Canada; 3 National Center for PTSD and Stanford University, 795 Willow Road NC-PTSD, Menlo Park, CA 94024 USA; 4 Department of Implementation Science, Wake Forest School of Medicine, 475 Vine Street, Winston-Salem, NC 27101 USA

**Keywords:** Adaptation, Evidence-based intervention, Implementation outcomes

## Abstract

**Background:**

Implementation science is shifting from qualifying adaptations as good or bad towards understanding adaptations and their impact. Existing adaptation classification frameworks are largely descriptive (e.g., who made the adaptation) and geared towards researchers. They do not help practitioners in decision-making around adaptations (e.g., is an adaptation likely to have negative impacts? Should it be pursued?). Moreover, they lack constructs to consider “ripple effects” of adaptations (i.e., both intended and unintended impacts on outcomes, recognizing that an adaptation designed to have a positive impact on one outcome may have unintended impacts on other outcomes). Finally, they do not specify relationships between adaptations and outcomes, including mediating and moderating relationships. The objective of our research was to promote systematic assessment of intended and unintended impacts of adaptations by using existing frameworks to create a model that proposes relationships among constructs.

**Materials and methods:**

We reviewed, consolidated, and refined constructs from two adaptation frameworks and one intervention-implementation outcome framework. Using the consolidated and refined constructs, we coded qualitative descriptions of 14 adaptations made to an existing evidence-based intervention; the 14 adaptations were designed in prior research by a stakeholder panel using a modified Delphi approach. Each of the 14 adaptations had detailed descriptions, including the nature of the adaptation, who made it, and its goal and reason. Using coded data, we arranged constructs from existing frameworks into a model, the Model for Adaptation Design and Impact (MADI), that identifies adaptation characteristics, their intended and unintended impacts (i.e., ripple effects), and potential mediators and moderators of adaptations’ impact on outcomes. We also developed a decision aid and website (MADIguide.org) to help implementation scientists apply MADI in their work.

**Results and conclusions:**

Our model and associated decision aids build on existing frameworks by comprehensively characterizing adaptations, proposing how adaptations impact outcomes, and offering practical guidance for designing adaptations. MADI encourages researchers to think about potential causal pathways of adaptations (e.g., mediators and moderators) and adaptations’ intended and unintended impacts on outcomes. MADI encourages practitioners to design adaptations in a way that anticipates intended and unintended impacts and leverages best practice from research.

Contributions to the literature

- Adaptation is a reality in implementation; implementers must adapt interventions to their context to improve intervention-context fit.
- Although frameworks exist for describing adaptations, they do not outline potential causal pathways, nor do they acknowledge the “ripple effects” adaptations can have (both intended and unintended impacts on outcomes).
- This paper contributes to the gaps in the literature by helping researchers and practitioners answer important questions around adaptation design (Should I do it? What outcomes might it impact, intended or unintended? How can I mitigate potential negative impacts?) and measurement (Which outcomes are likely to be impacted? What might causal pathways look like?).

## Background

Adaptations, or changes made to programs or interventions to align them with the context in which they are implemented [1, 4–6], are a reality in implementation. Adaptations allow for flexibility in implementation, improving the fit of an intervention with a new organization, population, or context. Adaptation is multi-faceted and dynamic; for a single intervention, several adaptations might be made. Adaptations can be made to interventions (i.e., the seven P’s—programs, practices, principles, procedures, products, pills, and policies [7]) and/or to the implementation strategies employed to facilitate the implementation of interventions. Moreover, a single adaptation may affect multiple outcomes, in both intended and unintended ways. For example, an adaptation intended to increase feasibility may have unintended “ripple effects,” such as decreasing fidelity or appropriateness.

The complex and multi-faceted nature of adaptations makes theories, models, and frameworks crucial to promote systematic, consistent adaptation descriptions and assessments. Although frameworks for adaptation exist and provide structure for classifying adaptations, they do not propose interrelationships among constructs or explanations of potential causal pathways like models and theories do [11]. The lack of models and theories hinders researchers from the prospective, predictive thinking needed to systematically test proposed relationships among constructs. The limitations of extant theories, models, and frameworks and lack of explanations of adaptations’ influence on outcomes are not surprising given that research on adaptation is still relatively young; only in recent years has the conversation shifted from fidelity versus adaptation (two opposing concepts) to balancing fidelity and adaptation (two complementary concepts) [4, 12–19].

One of the most comprehensive and most recently updated adaptation classification frameworks is the Framework for Reporting Adaptations and Modifications-Enhanced (FRAME) by Stirman and colleagues [2], which helps implementation scientists consistently describe the many facets of adaptations. FRAME is an updated version of Stirman et al.’s original framework [8] and includes additional considerations like (1) reason for adaptation (e.g., cultural/religious norms, time constraints, access to resources), (2) goal of the adaptation (e.g., increase reach, retention, feasibility, improve fit), (3) whether the adaptation was proactive or reactive (i.e., made proactively through a planning process that identifies ways to maximize fit and implementation success or made reactively during the course of program implementation, often due to unanticipated obstacles and in an impromptu manner), and (4) the adaptation’s relationship to core functions (i.e., whether the adaptation preserved fidelity to core functions of the original intervention).

Determining adaptations’ impact on outcomes represents a high priority objective in research [2]. However, FRAME does not distinguish among adaptations’ impact on implementation outcomes versus adaptations’ impact on service or patient outcomes [3], and because it measures goals for adaptation rather than actual outcomes, it does not distinguish between adaptations’ intended and unintended impacts (i.e., ripple effects). Similarly, process models for adaptation (e.g., [10, 35]) allow for iterative cycles that consider and measure outcomes, but they do not explicitly consider unintended impacts. Specifying and measuring a single intended goal of adaptation might not detect ripple effects of adaptations. We argue that systematic consideration of all potential impacts on a range of implementation and intervention outcomes is critical to adaptation.

The objective of our research was to (1) integrate outcomes into existing frameworks to promote a systematic assessment of adaptations’ intended and unintended impact on outcomes (i.e., ripple effects of adaptations), and (2) use existing frameworks to create an explanatory model that proposes possible interrelationships among constructs. By developing a model using existing frameworks to outline potential independent variables, mediators, moderators, and specific outcomes, we can move beyond the mere description of adaptations. In practice, this model can help practitioners think through adaptations’ influence on a range of outcomes to anticipate the intended and unintended impacts of adaptations. In research, this model can facilitate the systematic investigation of relationships between constructs—which characteristics of adaptations are predictive of which outcomes, under what circumstances, and why.

## Methods

To develop our model, we selected three frameworks from the literature: two adaptation frameworks [1, 2] and one outcome framework [3]. We selected FRAME [2] as the basis of our model because it is the most comprehensive and most recently updated adaptation classification framework. We selected Moore et al. and Proctor et al.’s work to meet our other two objectives: integrating implementation and intervention outcomes (addressed by Proctor et al.) and laying the foundation for investigating causal pathways between adaptation and outcomes (addressed by Moore et al.’s research which included explanations of adaptation characteristics’ relationship to outcomes, suggesting potential causal pathways) [1, 3].

AK, SB, and JM reviewed each framework for overlapping constructs/redundancies. We found substantial overlap between Moore et al.’s [1] constructs and FRAME [2]. With overlapping constructs identified, we then assessed, based on our own work adapting evidence-based practices and programs, whether any of the constructs should be consolidated or modified in any way. We made modifications to four constructs. See Table 1.

**Table 1:** Constructs from source frameworks, areas of overlap, modifications to existing frameworks, and reason for modifications

Construct and source framework (Stirman et al., Moore et al., or Proctor et al.)	Overlapping constructs	Modifications/additions to existing frameworks (in bold font)	Reason for modifications
**Stirman**: When did the modification occur? (Stirman) • Pre-implementation/planning/pilot • Implementation • Scale up • Maintenance/sustainment	None	None	N/A
**Stirman**: Were adaptations planned? • Planned/proactive (proactive) • Planned/reactive (reactive)	**Moore**: Timing • Whether the adaptation was proactive (occurring before implementation due to anticipated obstacles) or reactive (during implementation, due to unanticipated obstacles)	Were adaptations ***systematic/unsystematic and proactive/reactive?*** • **Systematic** and proactive • **Systematic** and reactive • **Non-systematic and proactive** • **Non-systematic** and reactive (modification)	Revised wording to remove concept of “planned” and replace with concept of “systematic.” This emphasizes the importance of how the adaptation was made (i.e., was it done using a systematic process), in addition to whether the adaptation was proactive (made due to an anticipated obstacle) or reactive (due to unanticipated challenges). This distinction is important as the assumption is that reactive adaptations are more likely to be non-systematic, impromptu, and less likely to be aligned with core functions of the intervention. However, our conceptualization allows for an understanding that reactive adaptations can still be made in a way that is systematic.
**Stirman**: Who participated in the decision to modify? • Political leaders, program leader, funder, administrator, program manager, intervention developer/purveyor, researcher, treatment/intervention team, individual practitioners, community members, recipients	None	None	N/A
**Stirman**: What was modified? • Content • Contextual • Training/evaluation • Implementation and scale-up activities	None	None	N/A
**Stirman:** At what level (for whom/what) is the modification made? • Individual, target intervention group, cohort/individuals that share a characteristic, individual practitioner, clinic/unit level, organization, network system/community	None	None	N/A
**Stirman**: Contextual modifications? • Format • Setting • Personnel • Population	None	None	N/A
**Stirman**: What is the nature of the content modification? • Tailoring/tweaking/refining, changes in packaging or materials, adding elements, skipping/removing elements, shortening/condensing (pacing/timing), lengthening/extending (pacing/timing), substituting, re-ordering, spreading, integrating into another framework, integrating into another treatment, repeating, loosening, departing from intervention followed by return to protocol (drift), drift without returning to protocol	None	None	N/A
**Stirman**: Relationship to core elements/fidelity? • Fidelity consistent/core elements preserved • Fidelity inconsistent/core elements changed • Unknown	**Moore:** Valence • Whether the adaptation aligned with the program’s theory/goals and thus were likely to have a positive/neutral/negative impact	Are adaptations aligned with **core functions**? • Fidelity consistent/core **functions** preserved • Fidelity inconsistent/core **functions** changed • Unknown	Removed references to “core elements” and instead focused on “core functions” due to recent publications in the literature which advocate for use of the term functions as it is the functions intervention components serve that is often core, not their exact form [20, 21]
**Stirman**: What was the goal? (Stirman) • Increase reach/engagement • Increase retention • Improve feasibility • Improve fit with recipients • Address cultural factors • Improve effectiveness/outcomes • Reduce cost • Increase satisfaction	**Moore:** Fit • Whether the adaptation was made for philosophical fit (to align with goals of organization or culture of target population) or logistical fit (to address mismatches in capacity, such as resources, time)	What was the *goal*? • **Improve likelihood of adoption** • Improve feasibility • Improve fit with recipients **(appropriateness)** o Address cultural factors • Increase satisfaction **(acceptability)** • Reduce cost • Increase reach/engagement **(penetration)** • **Improve fidelity** • Increase retention • **Improve sustainability** • Improve **intervention** effectiveness/outcomes • **No goal**	Added in/re-labeled additional mplementation outcomes (penetration, fidelity, sustainability, adoption, appropriateness, acceptability) to align this construct with Proctor’s framework. Also added in “no goal” for instances where an adaptation was made without a stated intended purpose or goal.
**Stirman**: Reasons for adaptation? • Sociopolitical (e.g., existing laws, political climate) • Organization/setting (e.g., available resources, competing demands) • Provider (e.g., race, first spoken languages, preferences, clinical judgement) • Recipient (e.g., race, access to resources, literacy, motivation/readiness)	None	None	N/A
**Proctor**: Implementation outcomes • Acceptability • Adoption • Appropriateness • Cost • Feasibility • Fidelity • Penetration • Sustainability	None	None	N/A
**Proctor**: Service/client outcomes (Proctor) • Service outcomes: efficiency, safety, effectiveness, equity, patient-centeredness, timeliness • Client outcomes: satisfaction, function, symptomatology	None	Called “intervention outcomes”	Re-labeled to provide an overarching category for both service and client outcomes.

The first construct we modified was FRAME’s “were adaptations planned?” (which overlapped with Moore’s construct of timing). The literature [1, 2, 13, 14, 18] often uses the terms “planned” and “proactive” (as well as unplanned and reactive) synonymously. In defining these terms, literature [1, 2, 13, 14, 18] unwittingly suggests an inextricable link between the impetus for the adaptation (i.e., whether the adaptation was proactive, meaning the adaptation was made in response to an anticipated obstacle, or whether the adaptation was reactive, meaning the adaptation was made in response to an unanticipated obstacle) and the process of adaptation (i.e., how systematic or unsystematic the process was). Literature [1, 2, 13, 14, 18] then implies that unplanned/reactive adaptations are more likely to be made using a haphazard process and thus more likely to detract from intervention core functions, resulting in negative impacts on outcomes. This conflation obfuscates the fact that reactive adaptations may still be made in a systematic way and proactive adaptations can be unsystematic. To acknowledge the diverse combinations of adaptation characteristics, we reconceptualized adaptations in terms of the degree to which they are systematic/unsystematic and proactive/reactive, removing the notion of planned/unplanned. This allows us to characterize, at the extremes of these continuous constructs, adaptations as follows:

- Systematic and proactive: made using a formal process that includes consulting data, theory, best practice, and/or stakeholders as well as considering the impact on outcomes (systematic), due to an anticipated obstacle/challenge (proactive). Examples of systematic, proactive adaptations could include those made using a formal process, prior to implementation (during planning/preparation/installation) [22, 23], due to anticipated areas of misfit between the intervention and context.
- Systematic and reactive: made using a formal process that includes consulting data, theory, best practice, and/or stakeholders as well as considering the impact on outcomes (systematic), due to an unanticipated obstacle/challenge (reactive). Examples of systematic and reactive adaptations could include those brought to light after implementation has begun via a quality improvement or Plan-Due-Study-Act cycle, but using a systematic process, or those made during program delivery by providers/practitioners in response to an unanticipated obstacle, but still using a systematic process.
- Unsystematic and proactive: made without a formal process (unsystematic), due to an anticipated obstacle (proactive). Examples of unsystematic, proactive adaptations may include those made at the individual level by a provider/practitioner prior to program delivery to address an anticipated barrier, such as patient literacy or language, but without consulting data/theory/stakeholders or considering the impact on outcomes.
- Unsystematic and reactive: made without a formal process (unsystematic), due to an unanticipated obstacle (reactive). Examples of unsystematic, reactive adaptations could include those made by a practitioner/provider improvising during delivery because of an unanticipated obstacle, but without consulting data/theory/stakeholders or considering the impact on outcomes.

Second, we modified FRAME’s construct of “relationship to core elements/fidelity?” (or valence in Moore’s framework) by removing reference to “core elements” and instead focusing solely on “relationship to core functions.” Recent publications [20, 21] argue for use of the term “core functions” and “forms” of interventions rather than previously used terminology of core components, core elements, and/or active ingredients [4, 13, 16, 17, 24–31]. This nomenclature emphasizes that it is the functions that intervention components serve that are “core,” not the exact form that they take, as it is possible to have several different forms that serve the same function. This modification is largely one of nomenclature, not a change in the meaning of the construct.

Third, we added potential goals of adaptation based on the literature [3] and our experience engaging in adaptation in the field. Several of our additions overlap with Proctor’s eight implementation outcomes as we often see in practice that implementers make adaptations to improve sustainability, fidelity, or other implementation outcomes. We also added “no goal”; this response option might apply to cases of unsystematic, reactive adaptations where the change was made without the goal of improving an outcome. Additional goals for adaptation included the following:

- Improve fidelity: to improve adherence, dose, or quality of intervention use (e.g., adding session checklists as reminders to improve adherence to protocols, adaptations to timing or mode of session to improve dose, and additional trainings, supervision, or coaching to improve quality).
- Improve sustainability: to improve maintenance or institutionalization of the intervention. Whereas adaptations to improve feasibility may increase initial/early intervention use, adaptations to improve sustainability may increase the intervention’s long-term use. For example, an intervention may be adapted to be delivered by existing staff to test initial feasibility and outcomes of the adapted intervention; over time, continuing to deliver the intervention along with regular duties may be deemed unsustainable for existing staff. Thus, the organization may later decide to further adapt the intervention to be delivered by newly hired staff, whose primary duties include intervention delivery. The initial adaptation was to improve feasibility; the second adaptation was made to improve sustainability. Timing of adaptations for feasibility vs sustainability may be driven by contextual factors; organizations that choose to adopt an intervention may adapt for initial feasibility early-on and re-adapt for sustainability after evaluating initial outcomes. Organizations that are mandated to adopt interventions may adapt for sustainability early-on as they know continuing the use of the intervention is required.
- Improve likelihood of adoption: to increase the likelihood that an intervention will be used at all. This is contrasted with adaptations that are made to improve feasibility or sustainability once the decision has been made to adopt the intervention. For example, an intervention that cannot be adapted to be used with an organization’s current electronic health record system may be a “non-starter” given the cost of a new electronic health record system. Adaptations to make the intervention compatible with the current electronic health record system are intended to improve adoption, not necessarily feasibility or sustainability.
- No goal: No intent. Whereas adaptations to improve acceptability are made to improve the perception that the intervention is agreeable, palatable, or satisfactory [3], those made with no goal would include those where there was no intent behind the adaptation (e.g., provider forgot a component or ran out of time). In these instances, the modification to the intervention was not intentional and had no specific goal in mind.

When considering the goal of the adaptation, an adaptation may have multiple intended goals. Thus, we encourage implementation scientists to consider the primary intention behind the adaptation and select multiple responses as needed, knowing that adaptations and the intent behind them may evolve as organizations and practitioners move through the phases of implementation.

Fourth, intervention outcomes include direct effects of the intervention itself. To account for this, we combined Proctor’s client and service outcomes into the broad construct “intervention outcomes.”

After reviewing constructs for redundancies and modifying construct definitions, we then used the revised constructs to develop our model. To develop our model, three authors (SB, AK, JM) coded 14 adaptations to an existing evidence-based intervention to improve the timeliness of referrals to hospice [34]. The evidence-based intervention was originally developed and tested in a randomized controlled trial to improve the timeliness of referrals to hospice for nursing home residents [34]. As part of a prior research effort [32], members of our research team (SB, AK) adapted the intervention from use in nursing home settings to use in home health settings. To design the adaptations, we engaged an expert panel comprised of 4 home health and hospice practitioners and used a modified Delphi approach where the expert panel reviewed the original intervention protocol and came to consensus on adaptations that would be needed to deliver the intervention in the new setting (home health). The stakeholder panel identified a total of 14 adaptations; major adaptations included changing eligibility criteria to make the intervention more appropriate for home health patients and multiple changes to delivery of the intervention to make the intervention more feasible to deliver as part of routine care (e.g., adapting who delivered the intervention, when, and how). From the modified Delphi discussions, we had rich descriptive data for each of the 14 adaptations, including who made the adaptation, descriptions of the adaptation, and the reason and intended goal of the adaptation. We used this descriptive data of each of the 14 adaptations and coded the data using the constructs from Table 1. Coding included structured responses (e.g., selecting what the nature of an adaptation was) as well as unstructured responses (reasons why we thought an adaptation might impact a certain outcome, and expected direction of the effect). SB, AK, and JM independently coded all 14 adaptations; afterwards, we met as a group to discuss and reconcile any discordant coding selections and how we might re-arrange constructs into domains to propose causal pathways of adaptations’ impact.

## Results

**Fig. 1:**
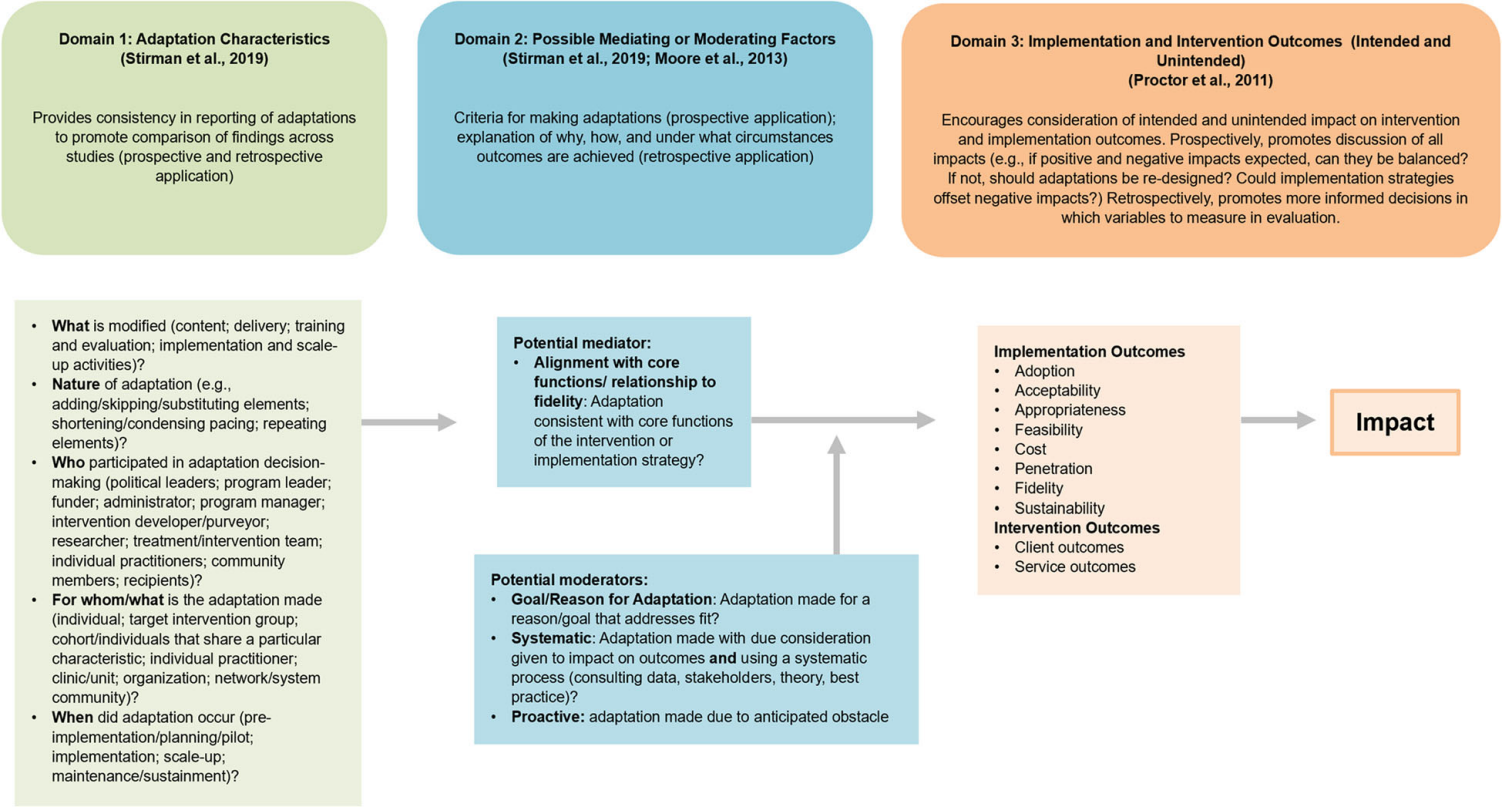
Model for Adaptation Design and Impact (MADI)

Our revised model, the Model for Adaptation Design and Impact (MADI), is presented in Fig. 1. MADI is organized into three domains (adaptation characteristics, possible mediating or moderating factors, and outcomes) and is designed for prospective use (i.e., during the process of deciding on and designing adaptations) and/or retrospective use (i.e., when adaptations are being implemented and evaluated).

### Domain 1: adaptation characteristics

Constructs in domain 1 include (1) what was modified, (2) the nature of the adaptation, (3) who participated in adaptation decision-making, (4) for whom/what the adaptation was made, and (5) when the adaptation occurred. These constructs help implementers classify adaptation attributes, providing an architecture that promotes consistency in reporting and comparison of findings across studies, which will help elucidate whether patterns emerge across studies. Constructs in domain 1 can be applied to adaptations made to interventions (i.e., the seven P’s—programs, practices, principles, procedures, products, pills, and policies [7]) or implementation strategies.

### Domain 2: possible mediating or moderating factors

Domain 2 comprises possible mediators or moderators of adaptation characteristics’ impact on outcomes, which we have categorized as such largely based on the findings of Moore et al.’s research. Moore et al. found that adaptations that were made systematically/proactively, were aligned with core functions, and were made to improve fit (i.e., had a stated goal/reason beyond logistics such as time or convenience) were more likely to incur positive impacts on outcomes [1]. Specifically, Moore et al.’s research showed that when adaptations were made with the goal of improving fit (e.g., to align the intervention with values of the target population), they were more likely to have a positive impact. Moore et al. also found that when adaptations were aligned with core functions of the intervention/the intervention’s theory of change, they were more likely to have a positive impact. This notion is supported by other literature that recommends all adaptations align with an intervention’s core functions [4, 10, 13, 16, 17, 24–29, 31, 33]. Finally, Moore found that adaptations that were made proactively were more likely to have a positive impact and more likely to be made with the goal of improving fit.

Thus, we believe Moore’s research presents initial empirical evidence that these constructs (goal of adaptation; whether adaptation was systematic; whether adaptation was proactive; whether adaptation aligned with core functions of intervention/implementation strategy) could be important moderators (as they can reverse, enhance, or diminish the impact on outcomes) and/or mediators (as some explain why an adaptation had certain impacts on outcomes). Although we believe the research is still too scant to say definitively which constructs are moderators and which are mediators and what interrelationships may exist, we suggest the following propositions that can be empirically tested in future research. First, we propose that alignment with core functions is a potential mediator of adaptations’ impact on outcomes because it preserves the mechanisms of change inherent to the intervention (or implementation strategy, depending on what is being adapted), thus preserving the link between intervention and outcome. We suspect the constructs of systematic, proactive, and goal of adaptation as potential moderators because they are aspects of the adaptation design and decision-making process that break or reverse the relationship between intervention and outcome, through relationship to core functions. Reactive adaptations are more likely to be made using an unsystematic process; an unsystematic process that does not consult theory, empirical data, or best practice, or anticipate impacts on outcomes, may compromise core functions. Similarly, adaptations with no stated goal may be less likely to have considered alignment with core functions, thus making the adaptation more likely to compromise outcomes. In short, we believe these constructs point to conditions under which adaptations (either to the intervention or implementation strategy) are more likely to have positive impacts on outcomes. Finally, the stated goal of the adaptation may also possibly be related to moderators of the original intervention itself. For example, an adaptation may be made due to patient motivation or comorbidity—these reasons for the adaptation may also be moderators of the intervention outcomes.

### Domain 3: implementation and intervention outcomes (intended and unintended)

We are ultimately interested in how adaptations impact both implementation and intervention outcomes. Thus, we included in domain 3 a range of possible implementation and intervention outcomes that adaptations could impact. In addition, we conceptualized outcomes in domain 3 as both intended and unintended outcomes because we know in practice that adaptations may have ripple effects from implementation to intervention outcomes (e.g., improved acceptability may diminish intervention effectiveness if the adaptation compromises core functions). Thus, when applying constructs in domain 3, we encourage implementers to consider both intended and unintended impacts.

### Prospective use of MADI

**Fig. 2:**
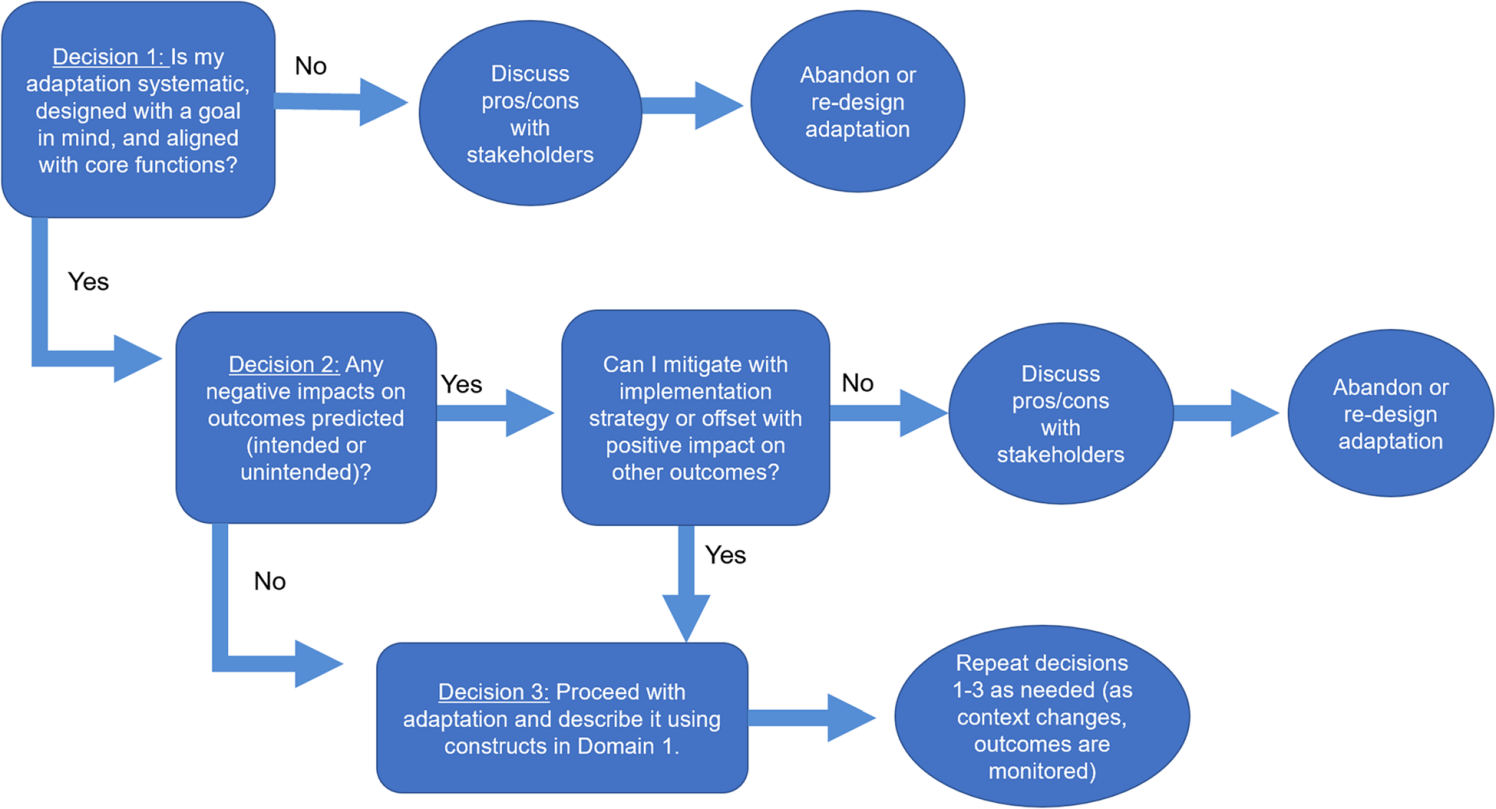
Decision-making guide for prospective use of MADI

We designed our model to be applied prospectively (during the process of deciding which adaptations to make) to avoid unintended impacts and/or retrospectively (after adaptations have been decided upon) to evaluate impacts of adaptations. Prospectively, our framework can guide decision-making around the design of adaptations and whether an adaptation should be made, based on potential impacts. See Fig. 2.

When using our model prospectively, we recommend using constructs in domain 2 as potential criteria in the decision-making process. Evidence suggests that non-systematic adaptations, those made without a goal in mind, and those that compromise core functions are associated with negative impact on outcomes [1]. Rating each potential adaptation using the four constructs in domain 2 can guide decision-making about whether to move forward with an adaptation as currently designed. If an adaptation is non-systematic, not aligned with core functions, or made without a clear goal, it may be more likely to negatively impact outcomes. In these instances, users should monitor outcomes closely and/or consider re-designing or abandoning the adaptation [35].

The second decision prompts users to consider each adaptation’s impact on all outcomes in domain 3, both intended and unintended. Based on our experience in practice, we suspect that adaptations may have unintended negative impacts in addition to the intended, positive impact and that, in these instances, there is no a priori clear answer about the impact of an adaptation. Despite this, we encourage users to engage in a discussion about the tradeoffs between potential impacts and use this discussion to help come to a consensus on whether to move forward with an adaptation, re-design it, or abandon it. Even if negative impacts are suspected, positive impacts on other outcomes may help “balance the scales” (i.e., even if negative impacts are suspect for one outcome, positive impacts on another outcome may result in a net-positive gain) and/or users may be able to develop implementation strategies to offset potential negative impacts. For example, if an adaptation designed to decrease costs is suspected to have an unintended, negative impact on acceptability, implementers may be able to “offset” the unintended negative impacts on acceptability by including an implementation strategy to increase buy-in as part of the implementation effort. In this instance, although an unintended negative impact is suspected, implementers may decide to move forward with the adaptation because they feel the implementation strategy to improve buy-in may be sufficient to mitigate unintended negative impacts. Although the success of this mitigation strategy is not guaranteed, implementers are, at a minimum, aware of the full range of impacts and actions they are taking to offset unintended impacts and can closely monitor outcomes during implementation. Thus, although MADI is not a predictive algorithm supported by empirical evidence, discussions guided by MADI can hopefully improve the status quo of making adaptations without considering their full range of impacts. In short, there may be instances where implementers decide to move forward with adaptations, despite anticipating negative, unintended impacts. This could happen for any number of reasons (e.g., legislative or funding requirements, leadership decisions). Whatever the reason for moving forward with an adaptation, engaging in thoughtful discussion with stakeholders about ripple effects can help implementers plan better for implementation (e.g., design implementation strategies to mitigate any negative impacts, anticipate outcomes that should be monitored in evaluation), hopefully increasing the likelihood of net-positive outcomes.

We recommend users document discussions surrounding decisions 1 and 2 to help guide the development of research questions and selection of variables to monitor in evaluation or other research efforts. We note that prospective application may be an iterative process, with a first step to decide whether to adapt the intervention at all or not, a decision point that can be supported by other decision aids [35]. Once researchers or practitioners have made the decision to adapt, our framework can be applied prospectively to aid in the design of adaptations that minimize negative impacts. For example, in a 2019 study [32], we adapted an intervention [34] designed to improve the timeliness of referrals to hospice in nursing homes for implementation in home health. As part of this effort, we also adapted the intervention from a randomized controlled trial model (where research staff delivered the intervention) to a practical model (where the intervention would be delivered by staff at the home health agency). As part of this shift, we adapted several aspects of the delivery of the intervention. Instead of research assistants carrying out the main activities of the intervention, home health staff would deliver the intervention as part of regular care. Although adaptations made to the delivery of the intervention were designed with the goal of improving feasibility and adoption, the adaptations also had the potential to compromise fidelity to the core functions of the intervention. The potential risk to fidelity was an unintended impact that we had to weigh against the potential benefit to feasibility and adoption—did the adaptations made to improve feasibility and adoption relate to core functions of the intervention and thus risk fidelity and effectiveness? These potential “ripple effects” of our adaptations were discussed and considered proactively by our research team before deciding whether to move forward with the adaptations. Because we anticipated potential negative impacts if fidelity were compromised, it was an outcome we knew we would have to closely monitor when testing the adapted intervention; it was an unintended impact we were aware of and able to monitor in evaluation [32].

Finally, although we encourage practitioners to engage in adaptation (and apply our model/decision aid) prior to implementation, as early as the planning/preparation/installation phases [22, 23], we note that MADI can be applied throughout implementation and advocate for continued application, as adaptation is an inherently iterative process. Because adaptations are designed to improve the fit of the intervention with context, adaptation is not a “one and done” process as context is continually changing and outcomes are, ideally, continually monitored to inform improvements. The continuous evolution of adaptations as implementation progresses is increasingly acknowledged in the literature (e.g., [36, 37]) and underscores the importance of considering adaptation as an iterative, ongoing process. Thus, although implementers may initially use MADI in the planning/design phase to proactively and systematically design adaptations prior to implementation, we encourage continuous use of MADI as implementation progresses and teams monitor impacts on outcomes.

### Retrospective use of MADI

**Fig. 3:**
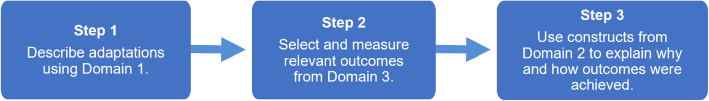
Retrospective application of MADI

Users can apply our model as a scaffolding for evaluating research questions and/or selecting variables and for testing hypotheses and assumptions about causal pathways. See Fig. 3.

If an intervention has already been adapted and researchers aim to evaluate impacts of the adapted intervention, MADI can be used as a discussion guide to help determine potential mediators/moderators and which array of outcomes may be impacted by which adaptations. Because adaptations can have ripple effects (i.e., both intended and unintended impacts), using our framework to guide discussion can help ensure that outcome variables are appropriately selected. For example, if researchers are designing an evaluation of an adapted intervention, they should first describe the adaptations using the constructs from MADI’s domain 1 (e.g., who made the adaptation and when, nature of the adaptation). These characteristics of adaptations may be used as independent variables in evaluation. Second, researchers could then use constructs in domain 3 to consider both the intended and unintended impacts of adaptations. Traditionally, researchers may choose outcome variables to measure in an evaluation based on the intended impact of the adaptation; however, this may result in a biased selection of outcome variables for research as it only captures a subset of adaptations’ impacts. Our own practice shows us that adaptations can have multiple intended and unintended impacts (ripple effects); thus, researchers may benefit from a robust discussion of each outcome in domain 3 when choosing outcome variables to include in evaluation. Such discussion may allow for more robust analyses that can capture the intended and unintended impacts of adaptations. Finally, researchers could use constructs in domain 2 to determine potential mediators and moderators to include in evaluation. By noting whether adaptations align with core functions, were made with an intended goal, and were systematic and proactive, researchers can assess causal pathways of adaptations.

## Discussion

A primary goal in research on adaptation is building the knowledge base on what types of adaptations impact which outcomes under which circumstances and why. Classification frameworks (e.g., [1, 2]) help us classify adaptations but do not integrate outcomes, nor do they specify interrelationships or potential causal pathways among constructs. Similarly, other reviews [9] investigate adaptations, reasons, and outcomes, but do not posit relationships among constructs. We integrated and updated three existing frameworks by turning these frameworks into a model, MADI, which outlines possible interrelationships for how adaptations influence outcomes.

### Components of MADI

MADI elaborates on FRAME and other frameworks in two ways. First, FRAME alluded to outcomes (the goal construct states the intent of the adaptation, which implicates outcomes). MADI compels users to consider how adaptations may influence all outcomes listed in our outcome domain and whether those outcomes are intended or unintended; we have found in practice that adaptations often have unintended ripple effects on multiple outcomes, even if they were designed with the intent of having a positive impact on one outcome in particular. Second, MADI arranges constructs from FRAME and other frameworks into an explanatory model that shows possible interrelationships for how adaptations influence outcome as MADI elucidates potential mediating and moderating relationships for how adaptations influence outcomes. In this sense, MADI advances the field by moving from a framework (a structure or overview of descriptive categories that does not provide explanations [11]) to an explanatory model that proposes explanations of potential mediating and moderating relationships underlying adaptations’ impact on outcomes.

### How MADI advances implementation science

Our model builds on the existing literature and moves the adaptation literature base forward in several ways. First, unlike other models which simply categorize adaptations (e.g., FRAME), aid in the decision of whether to adapt at all (e.g., [35]), or provide guidance on the process of adaptation (e.g., [5, 38]), our model aids in the design of adaptations and adaptation-related research questions, emphasizing the intended and unintended consequences that adaptations can have, helping practitioners minimize negative impacts, and helping researchers systematically evaluate them. We hope that MADI and the accompanying decision aid will encourage practitioners to think about adaptation early and engage in a systematic decision-making process and that it will encourage ongoing adaptation design conversations throughout the implementation lifecycle.

Second, MADI directs attention to potential impacts and ripple effects in the adaptation decision-making process. This contrasts with current literature, which encourages focusing on whether core functions are compromised as the primary (and often singular) criterion for designing potential adaptations. We argue that, to achieve desired outcomes, in addition to assessing alignment with core functions, potential adaptations must avoid compromising implementation. Indeed, adaptations are often proposed to facilitate positive impacts on implementation outcomes. Adaptations that are made to improve one implementation outcome (e.g., acceptability) may compromise another implementation outcome (e.g., feasibility), which may, in turn, compromise the intervention’s overall impact.

Finally, we believe that MADI will help implementation researchers build and investigate research questions using a common taxonomy, which will more quickly move the field towards an empirically validated middle-range theory of adaptation, as it will allow for common nomenclature across studies. We hope researchers will use domains from MADI and select constructs to build specific theories of change for their interventions and adaptations. In this sense, although MADI is a model because it highlights potential interrelationships, it can be used like a framework to guide research as it provides a “menu of constructs” for researchers to select from when building potential causal pathways that can then be empirically tested through research. MADI will provide researchers a consistent, systematic way to select and describe potential mediators, moderators, and outcomes for research studies, which we hope facilitates the testing of mechanisms of change underlying adaptations to develop empirically valid theories of change. The next steps are to test hypotheses and pathways, with the goal of developing a theory of change related to adaptations.

To promote dialogue in the field, we have developed a website for MADI (MADIguide.org). We envision this website being a hub for MADI and housing several resources available to implementation scientists. Specifically, we have developed a comprehensive MADI Application and Discussion guide, which is available on the MADIguide.org website. This guide walks researchers and practitioners through applying MADI in their work and includes step-by-step instructions and discussion questions, as well as examples of how MADI has been applied already in research. We encourage implementation scientists to visit the MADIguide.org website to access the MADI Application and Discussion guide and other resources.

### Limitations and scope of MADI

Our model has several limitations. First, although it is evidence-informed (i.e., builds on existing, widely accepted and adopted frameworks) and was constructed using a systematic process (i.e., the coding of adaptation data), it has not been empirically tested. This means that although we suspect constructs in domain 2 may be important mediators or moderators, we have not empirically tested this model to validate these relationships. Moreover, we have not tested whether the outcomes in domain 3 are the most common or most important outcomes likely to be impacted by adaptation. Despite this, we believe that our model is unique in its comprehensive nature and, as such, represents an important step forward for developing and testing theories of adaptation.

Although we believe MADI adds value to the field, we also acknowledge MADI’s limited scope in comparison with the larger adaptation process. MADI is not intended to be an adaptation process framework (i.e., a framework that outlines step-by-step instructions for the full lifecycle of the adaptation process [10]), as it does not cover all steps in the adaptation process. For example, once an intervention has been selected, adaptation is a process that generally consists of (1) understanding the intervention (identifying its core functions and forms; reviewing related materials, such as protocols or manuals); (2) identifying and consulting with experts and stakeholders throughout the adaptation process to better understand the new setting where the adapted intervention will be implemented and consulting with original intervention developers as needed to seek advice on adaptation; (3) identifying differences between the context the intervention was designed for and the new context, which will inform potential areas of misfit; (4) identifying aspects of the original intervention that need to be adapted; (5) designing and specifying adaptations; (6) conducting any feasibility testing of the adapted intervention (pre-testing/pilot testing and making further adaptations if necessary); (7) training staff on the adapted intervention; (8) implementing the adapted intervention; and (9) evaluating the impact of the adapted intervention [10].

In practice, MADI is most applicable in step 5, designing adaptations. As such, MADI assumes that implementers have already completed several important preceding steps in the adaptation process, including identifying core functions (underlying principles that make the intervention effective and, thus, should not be adapted [21]) and forms of the intervention, identifying and consulting with relevant stakeholders and experts, identifying context differences, and deciding what needs to be adapted. In research, MADI is helpful in step 9, designing the evaluation of an adapted intervention. When applied in research, MADI assumes that prior steps in the adaptation process have been completed according to best practice, including identifying core functions and forms of the intervention, consulting stakeholders and experts, and clearly specifying all characteristics of adaptations.

Whether applying MADI in research or practice, if preceding steps in the adaptation process have not been completed, we recommend implementation scientists integrate other approaches or frameworks into their efforts to ensure these steps are completed before applying MADI. In particular, we underscore the importance of identifying core functions and forms of existing interventions before engaging in any adaptation efforts. For further guidance on identifying core functions and forms, a key step in the adaptation process to preserve the effectiveness of the original intervention, we recommend reviewing [20, 21]. In addition, for implementation scientists adapting an existing intervention, it may be helpful to pair MADI with an existing adaptation process framework to promote a systematic, comprehensive, evidence-informed approach to adaptation. We recommend the scoping review by Escoffery et al. [10] for a comprehensive listing of adaptation process frameworks for adapting public health interventions.

## Conclusion

Future research is needed to test our model and confirm mediating and moderating relationships, as well as impacts on outcomes. In addition, more applied work is needed to assess the relevance and feasibility of using MADI to aid in decision-making around adaptation design. Finally, although we acknowledge that adaptations can be made to interventions or implementation strategies, our model does not discern different mediating/moderating pathways and different impacts on outcomes based on what is being adapted (implementation strategy or intervention). More research and application of MADI is needed to determine whether there are different pathways to outcomes based on what is being adapted (i.e., intervention or implementation strategy). We hope MADI can serve as a springboard for promoting systematic discussion among adaptation decision-makers and provide scaffolding for building the evidence-base around mechanisms of change underlying adaptations.

## Data Availability

The datasets used and/or analyzed during the current study are available from the corresponding author on reasonable request.

## Abbreviations

FRAME: Framework for Reporting Adaptations and Modifications-Enhanced
IDEA: Iterative Decision-making for Evaluation of Adaptations
MADI: Model for Adaptation Design and Impact

## References

[1] Moore JE, Bumbarger BK, Cooper BR (2013). Examining adaptations of evidence-based programs in natural contexts. J Prim Prev.

[2] Stirman SW, Baumann AA, Miller CJ (2019). The FRAME: an expanded framework for reporting adaptations and modifications to evidence-based interventions. Implement Sci.

[3] Proctor E, Silmere H, Raghavan R, Hovmand P, Aarons G, Bunger A (2011). Outcomes for implementation research: conceptual distinctions, measurement challenges, and research agenda. Adm Policy Ment Health Ment Health Serv Res.

[4] Castro FG, Barrera M, Martinez CR (2004). The cultural adaptation of prevention interventions: resolving tensions between fidelity and fit. Prev Sci.

[5] Lee SJ, Altschul I, Mowbray CT (2008). Using planned adaptation to implement evidence-based programs with new populations. Am J Community Psychol.

[6] Solomon J, Card JJ, Malow RM (2006). Adapting efficacious interventions: advancing translational research in HIV prevention. Evaluation & the health professions.

[7] Brown CH, Curran G, Palinkas LA, Aarons GA, Wells KB, Jones L (2017). An overview of research and evaluation designs for dissemination and implementation. Annu Rev Public Health.

[8] Stirman SW, Miller CJ, Toder K, Calloway A (2013). Development of a framework and coding system for modifications and adaptations of evidence-based interventions. Implement Sci.

[9] Escoffery C, Lebow-Skelley E, Haardoerfer R, Boing E, Udelson H, Wood R (2018). A systematic review of adaptations of evidence-based public health interventions globally. Implement Sci.

[10] Escoffery C, Lebow-Skelley E, Udelson H, Böing EA, Wood R, Fernandez ME (2019). A scoping study of frameworks for adapting public health evidence-based interventions. Transl Behav Med.

[11] Nilsen P (2015). Making sense of implementation theories, models and frameworks. Implement Sci.

[12] Allen JD, Linnan LA, Emmons KM, Brownson R, Colditz G, Proctor E. Fidelity and its relationship to implementation effectiveness, adaptation, and dissemination. Dissemination and implementation research in health: Translating science to practice. 2012:281–304.

[13] Carvalho ML, Honeycutt S, Escoffery C, Glanz K, Sabbs D, Kegler MC (2013). Balancing fidelity and adaptation: implementing evidence-based chronic disease prevention programs. J Publ Health Manag Pract.

[14] Lendrum A, Humphrey N, Greenberg M. Implementing for success in school-based mental health promotion: the role of quality in resolving the tension between fidelity and adaptation. In: Mental Health and Wellbeing through Schools. Routledge; 2016. p. 77-87.

[15] Aarons GA, Green AE, Palinkas LA, Self-Brown S, Whitaker DJ, Lutzker JR (2012). Dynamic adaptation process to implement an evidence-based child maltreatment intervention. Implement Sci.

[16] Bopp M, Saunders RP, Lattimore D (2013). The tug-of-war: fidelity versus adaptation throughout the health promotion program life cycle. J Prim Prev.

[17] Van Daele T, Van Audenhove C, Hermans D, Van den Bergh O, Van den Broucke S (2014). Empowerment implementation: enhancing fidelity and adaptation in a psycho-educational intervention. Health Promot Int.

[18] Kemp L (2016). Adaptation and fidelity: a recipe analogy for achieving both in population scale implementation. Prev Sci.

[19] Larsen T, Samdal O (2007). Implementing second step: balancing fidelity and program adaptation. J Educ Psychol Consult.

[20] Jolles MP, Lengnick-Hall R, Mittman BS (2019). Core functions and forms of complex health interventions: a patient-centered medical home illustration. J Gen Intern Med.

[21] Kirk MA, Haines ER, Rokoske FS, Powell BJ, Weinberger M, Hanson LC, Birken SA: A case study of a theory-based method for identifying and reporting core functions and forms of evidence-based interventions. Translational Behavioral Medicine 2019. 10.1093/tbm/ibz178 PMC7877297 31793635

[22] Aarons GA, Hurlburt M, Horwitz SM (2011). Advancing a conceptual model of evidence-based practice implementation in public service sectors. Adm Policy Ment Health Ment Health Serv Res.

[23] Metz A, Bartley L (2012). Active implementation frameworks for program success. Zero to Three.

[24] Elliott DS, Mihalic S (2004). Issues in disseminating and replicating effective prevention programs. Prev Sci.

[25] Mowbray CT, Holter MC, Teague GB, Bybee D (2003). Fidelity criteria: development, measurement, and validation. Am J Eval.

[26] Schoenwald SK, Hoagwood K (2001). Effectiveness, transportability, and dissemination of interventions: what matters when?. Psychiatr Serv.

[27] Botvin GJ (2004). Advancing prevention science and practice: challenges, critical issues, and future directions. Prev Sci.

[28] Morrison DM, Hoppe MJ, Gillmore MR, Kluver C, Higa D, Wells EA (2009). Replicating an intervention: the tension between fidelity and adaptation. AIDS Education & Prevention.

[29] Cohen DJ, Crabtree BF, Etz RS, Balasubramanian BA, Donahue KE, Leviton LC (2008). Fidelity versus flexibility: translating evidence-based research into practice. Am J Prev Med.

[30] Blase K, Fixsen D: Core intervention components: identifying and operationalizing what makes programs work. ASPE Research Brief. US Department of Health and Human Services 2013.

[31] Rabin, B. Fidelity and adaptation for implementation science: how can we reconcile the tension?. In*.* Center for Research in Implementation Science and Prevention (CRISP) Seminar Series: University of Colorado Anschutz Medical Campus; 2016.

[32] Kirk MA, Hanson LC, Weinberger M, Haines ER, Rokoske FS, Powell BJ (2019). Pilot test of an adapted intervention to improve timeliness of referrals to hospice and palliative care for eligible home health patients. J Palliat Med.

[33] Marques L, Valentine SE, Kaysen D, Mackintosh M-A, De Silva D, Louise E (2019). Provider fidelity and modifications to cognitive processing therapy in a diverse community health clinic: associations with clinical change. J Consult Clin Psychol.

[34] Casarett D, Karlawish J, Morales K, Crowley R, Mirsch T, Asch DA (2005). Improving the use of hospice services in nursing homes: a randomized controlled trial. Jama.

[35] Miller CJ, Wiltsey-Stirman S, Baumann AA. Iterative decision-making for evaluation of adaptations (IDEA): a decision tree for balancing adaptation, fidelity, and intervention impact. Journal of Community Psychology. 2020. 10.1002/jcop.22279 PMC7261620 31970812

[36] Izumi S, Caron D, Combe A, Archambault P, Shankle N, Michaels L, Dorr D, Totten A: Adaptation and fidelity of serious illness care program across primary care settings in the US and Canada. In: 12 th Annual Conference on the Science of Dissemination and Implementation: 2019: AcademyHealth; 2019.

[37] Yakovchenko V, Chinman M, Gonzalez R, Park A, Morgan T, Chartier M, Ross D, Rogal S: Longitudinal assessment of expert recommendations for implementing change (ERIC) strategies in the uptake of evidence-based practices for hepatitis c treatment in the veterans administration: year four. In: 12 th Annual Conference on the Science of Dissemination and Implementation: 2019: AcademyHealth; 2019.

[38] Bartholomew Eldredge L, Highfield L, Hartman M, Mullen P, Leerlooijer J, Fernandez M, Bartholomew Eldredge L, Markham C, Ruiter R, Fernandez M, Kok G, Parcel G (2016). Using intervention mapping to adapt evidence-based interventions. Planning health promotion programs: an intervention mapping approach.

